# Design, Synthesis, and Biological Activity of New Triazole and Nitro-Triazole Derivatives as Antifungal Agents

**DOI:** 10.3390/molecules22071150

**Published:** 2017-07-10

**Authors:** Hossein Sadeghpour, Soghra Khabnadideh, Kamiar Zomorodian, Keyvan Pakshir, Khadijeh Hoseinpour, Nabiollah Javid, Ehsan Faghih-Mirzaei, Zahra Rezaei

**Affiliations:** 1 Department of Medicinal Chemistry, School of Pharmacy, Shiraz University of Medical Sciences, Shiraz 7146864685, Iran; sadeghpurh@sums.ac.ir (H.S.); khabns@sums.ac.ir (S.K.); hoseinpour65k@gmail.com (K.H.); javidn@yahoo.com (N.J.); 2 Department of Medical Mycology and Parasitology, School of Medicine, Shiraz University of Medical Sciences, Shiraz 7134845794, Iran; zomorodian@sums.ac.ir (K.Z.); pakshirk@sums.ac.ir (K.P.); 3 Department of Medicinal Chemistry, School of Pharmacy, Kerman University of Medical Sciences, Kerman 7616911319, Iran; ehsancontact@kmu.ac.ir

**Keywords:** fluconazole, synthesis, antifungal, lanosterol 14α-demethylase, docking, nitrotriazole

## Abstract

In this study two series of fluconazole derivatives bearing nitrotriazole (series A) or piperazine ethanol (series B) side chain were designed and synthesized and then docked in the active site of lanosterol 14α-demethylase enzyme (1EA1) using the Autodock 4.2 program (The scripps research institute, La Jolla, CA, USA). The structures of synthesized compound were confirmed by various methods including elemental and spectral (NMR, CHN, and Mass) analyses. Then antifungal activities of the synthesized compound were tested against several natural and clinical strains of fungi using a broth microdilution assay against several standard and clinical fungi. Nitrotriazole derivatives showed excellent and desirable antifungal activity against most of the tested fungi. Among the synthesized compounds, **5a**–**d** and **5g**, possessing nitrotriazole moiety, showed maximum antifungal activity, in particular against several fluconazole-resistant fungi.

## 1. Introduction

Fungal infections have become a serious clinical challenge in recent years due to clinical issues such as cancer chemotherapy, transplantation, HIV infections, and the use of immunosuppressive agents [1,2,3,4]. Among the antifungal agents, azoles were used widely in treatment of fungal infections, which inhibit the lanosterol 14α-demethylase (CYP51) to prevent ergosterol biosynthesis from lanosterol in yeast and fungi. The selective inhibition of CYP51 causes depletion of ergosterol and the accumulation of lanosterol and other 14α-methyl sterols, resulting in the growth inhibition of fungal cells [5]. Fluconazole, a triazole derivative, is a first-line antifungal agent with low toxicity and high antifungal activity. Some species of fungi are resistant against fluconazole, and this is now becoming a serious clinical problem [5]. In order to enhance the antifungal activity or reduce antifungal resistance, many fluconazole derivatives were synthesized containing other heterocycles instead of 1,2,4-triazole [6,7,8,9,10,11,12,13], such as tetrazole [7], indole [8], indoline, benzimidazole [9], imidazole or 1,2,3-triazol [12,13]. Voriconazole and ravuconazole are two clinically important antifungal triazoles in which the second ring is replaced by fluoropyrimidine and thiazole derivatives, respectively [14]. The introduction of halogenated quinazoline instead of a triazole ring in the fluconazole structure results in albaconazole with higher in vitro activity in comparison to fluconazole [6]. Antifungal activity improved in the amide derivatives containing a pyrrolo-pyridine portion [6]. The triazole containing agents can show various biological activities other than antifungal ones [15,16]. Among them, the cytotoxic effects can lead us to new antineoplastic hits [17]. On the other hand some studies indicated that triazole-functionalized multi-walled carbon nanotubes can be used to remove metal impurities from water [18] or can be used as an effective antibiofouling agents [19].

There are a few reports about the antifungal activity of nitroimidazole or nitropyrazole [20]. To our knowledge, there is no report about the antifungal activities of nitrotriazoles. On the other hand introducing a piperazine ring to the azole structure leads to better antifungal activity and fewer toxicities [7,21]. Itraconazole and posaconazole with a piperazine ring in their structures are the best examples [6]. Fluconazole derivatives with substituted phenoxypropyl piperazine side chains or a piperazinyl phenyl side chain showed in vitro antifungal activity against several pathogenic fungi [10,11,21,22].

Docking simulation is a key tool in structural molecular biology and computer-assisted drug design (CAAD) to predict the “best-fit” orientation of ligands against active sites in antifungal discovery [11,23,24].

In this study, two series of fluconazole derivatives bearing a nitrotriazole or piperazine ethanol side chain were designed and then docked in the active site of 14α-demethylase enzyme (1EA1) using the Autodock 4.2 program. Designed compounds with the least final docking energy (FDE) values were synthesized and their antifungal activities were tested against several yeasts and filamentous fungi.

## 2. Results and Discussion

### 2.1. Chemistry

**Scheme 1:**
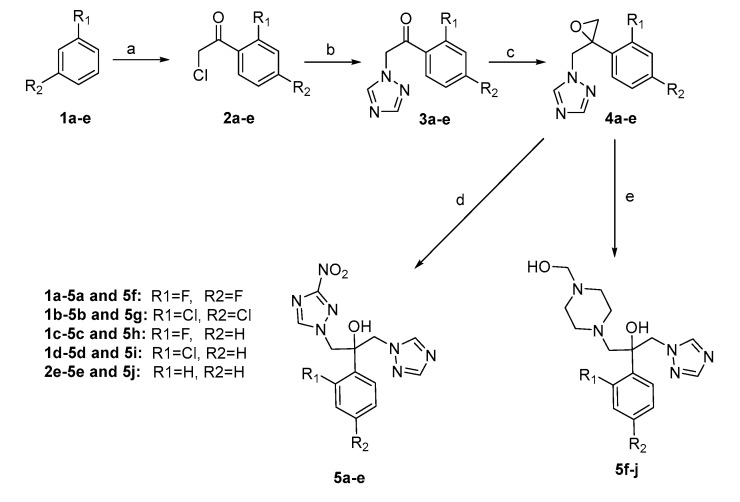
Synthesis of the new fluconazole compounds; conditions: (**a**) AlCl_3_, 1,2-dichloroethane, chloroacetyl chloride, 25–30 °C; (**b**) 1H-1,2,4-triazole, NaHCO_3_, toluene, reflux; (**c**) trimethylsulfoxonium iodide (TMSI), NaOH, tetraethylammonium bromide (TEAB), dichloromethane, toluene, 60 °C; (**d**) EtOH, Et_3_N, 3-nitro-1,2,4 triazol, 80 °C; (**e**) EtOH, Et_3_N, 2-(piperazin-1-yl) ethanol, 80 °C.

In this study, two series of compounds, series A (**5a**–**e**), which contain nitrotriazole, and series B (**5f**–**j**), with piperazine moiety in their structures, were synthesized (Scheme 1). The synthesis routes are based on the previous reports in the literatures [8,10,11,12,13,21,25] with few modifications. Briefly, the key oxirane intermediate (compound **4**) was synthesized using trimethylsulfoxonium iodide (TMSI) and sodium hydroxide in dichloromethane/toluene in the presence of a phase transfer catalyst, tetraethylammonium bromide (TEAB). In the final step, 3-Nitro-1,2,4-triazole or 2-(1-pyperazinyl) ethanol was allowed to react with epoxy intermediate 4. 3-Nitro-1,2,4-triazole or 2-(1-pyperazinyl) ethanol could be added to compound **2** or epoxy intermediate **4**. Based on our experiences the goal products **5a**–**j** were obtained in higher yields when 3-nitro-1*H*-1,2,4-triazole or 2-(1-pyperazinyl) ethanol were applied in the final step.

### 2.2. Biological Activity

The antifungal activities (MICs) of the synthesized compounds were investigated against several standard and clinically isolated fungi using the serial microdilution assay. Fluconazole was used as the positive control (Table 1 and Table 2). The MICs showed that yeasts are more sensitive than filamentous fungi to the synthesized compounds. Antifungal activity of fluconazole against tested fungi showed that fluconazole has also minimal activity on filamentous ones. These findings suggested that the mechanism of action of the new compounds may be similar to fluconazole. More interestingly, the clinically isolated microorganisms were more sensitive than standard species (Table 1). The results showed that all the synthesized derivatives except **5i**, **5j** and **5h** exhibited moderate to high in vitro antifungal activities. Compounds **5a**, **5b** and **5g**, which bear 2, 4-dichlorophenyl or 2,4-difluorophenyl group, had better antifungal activity against most tested fungal strains (Table 1 and Table 2). Compound **5b** had the most antifungal activity against all fungal strains except *Aspergillus* sp. As shown in Table 1 compounds **5b**, **5f**, **5g** and **5i** had antifungal activity against fluconazole-resistant fungi (*C. krusei* (ATCC 6258)). **5b** also showed antifungal activity more than fluconazole on some standard and clinically isolated strains such as *C. albicans* (CBS 5982), *C. albicans* (CBS 1905), and *C. krusei* (ATCC6258). The most sensitive fungal strains to the synthesized compounds were *C. dubliniensis* (CBS 8501), *C. dubliniensis* (CBS 7987), *C. dubliniensis* (CBS 7988), *C. albicans* (CBS 1912), and *C. albicans* (CBS 1905), whereas the *Aspergillus* species were not sensitive to any of our compounds.

### 2.3. Docking

Fluconazole and all of the synthesized compounds **5a**–**j** were docked into the active site of lanosterol 14α-demethylase enzyme (1EA1) and the FDE was calculated (Table 3). As shown in Table 2, all compounds had more negative FDE values than fluconazole, and compound **5b** had the best FDE value. **5b** had the highest LogP (Log Partition Coefficient) and the best FDE as well as overall highest antifungal activity. The results revealed some correlation between these parameters and antifungal effects (Table 1, Table 2 and Table 3). Generally, the synthesized compounds in series A had more negative FDE and better antifungal activities compared to series B. This may be attributed to the better electrostatic interaction of NO_2_ moiety of these compounds with Fe^2+^ in the heme molecule of 14α-demethylase. In addition, the compounds in series A had higher log P compared to series B. As more lipophilic agents demonstrated better antifungal activity, it can be concluded that lipophilicity can enhance the penetration of these compounds to fungal cells.

### 2.4. Structure Activity Relationship

**Figure 1:**
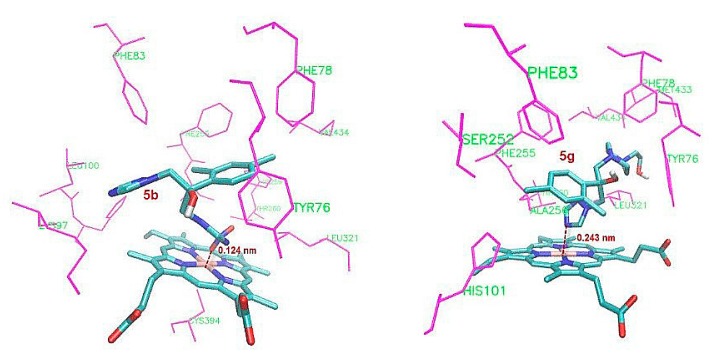
Visualizing the active site in the presence of ligands: left (**5b**), right (**5g**).

The replacement of one of the triazole rings with nitrotriazole leads to increased antifungal activity for several synthesized compound in compare with fluconazole. The binding model of compound **5b** and **5g** with 14α-demethylase is illustrated in Figure 1. As shown in Figure 1, the molecules are well accommodated in the active pocket. The nitrotriazole derivatives, series A, had the better electrostatic interaction with the heme core of enzyme in comparison with piperazine derivatives, series B. The iron-N distance in the active site of 14α-demethylase for **5b** and **5g** (0.124 nm vs. 0.243 nm) may be responsible for this interaction leading to better enzyme inhibition and antifungal activity. These findings indicated that the nitro group may be able to provide better interaction in the active site of 14α-demethylase in comparison with N_4_ of triazole (Figure 1). Among series A, compound **5b** showed the best antifungal effect; in series B, **5g** had the most antifungal effect against several tested microorganisms. These two compounds had two chlorine in their structures. The elimination of halogens from compounds to form compounds **5e** and **5j** reduced antifungal activity. This can be attributed to the better hydrophobic interaction of electron-poor phenyl group with a phenyl ring of Phe83, Phe78, and Phe255, which surround the ligand in the active site (Figure 1). On this basis the replacement of chlorine with fluorine or hydrogen will result in less antifungal effect and FDE (Table 1 and Table 2).

## 3. Materials and Methods

### 3.1. Chemistry

All reagents and solvents were purchased from commercial suppliers and used without further purification. Melting points were determined by using an Electrothermal 9100 digital melting point apparatus (Electrothermal, Essex, UK). The reaction progress and purity of the synthesized compounds were checked on Merck aluminum plates precoated with silica gel 60 F-254 (Merck KGaA, Darmstadt, Germany). UV radiation and/or iodine were used as the visualizing agents. Silica gel column chromatography was performed with Silica gel 60G (Merck KGaA, Darmstadt, Germany). Spectroscopic data were recorded on the following instruments: ^1^H-NMR spectra was recorded on a Bruker 500 spectrometer with TMS (tetramethylsilane) (Germany) as internal standard and CDCl_3_ as the solvent and ^13^C-NMR spectra was recorded on a Bruker 300 spectrometer (Germany). Chemical shifts (δ values) and coupling constants (*J* values) are given in ppm and Hz, respectively. Signal multiplicities are represented by s (singlet), d (doublet), t (triplet), dd (double of doublet), dt (double of triplet), m (multiplet), and brs (broad singlet). Mass spectra were recorded on a Hewlett-Packard GC-MS (Agilent, Santa Clara, CA, USA). The CHN analysis were done by ECS 4010 (Costech Company, Firenze, Italy).

#### 3.1.1. General Procedure for the Synthesis of 2-Chloro-1-(2,4-disubstituted phenyl) Ethanone (**2a**–**e**)

Disubstituted benzenes **1a**–**e** (50 mmol) and anhydrous aluminum chloride (60 mmol, 7.98 g) were stirred in 60 mL dichloroethane at 30 °C for 30 min. The mixture was then cooled to 0 °C in an ice bath and was charged dropwise with chloroacethyl chloride solution (54 mmol, 6.21 g) while stirring during 30 min. Stirring was continued for further 30 min keeping the reaction mixture at 0–10 °C. Then the mixture was warmed to room temperature and stirred for 24 h. Afterward the reaction was quenched by the addition of hydrochloric acid (1 M) and the mixture was extracted with dichloroethane in 0–5 °C. The combined organic layers were washed with 2 × 20 mL saturated NaHCO_3_ and then with cold water and brine, dried over anhydrous sodium sulfate (Na_2_SO_4_) and concentrated in a vacuum. The crude product was purified by recrystallization from n-hexane, affording **2a**–**e**.

#### 3.1.2. General Procedure for the Synthesis of 1-(2,4-Disubstituted phenyl)-2-(1*H*-1,2,4-triazol-1-yl) Ethanone (**3a**–**e**)

A flask equipped with a reflux condenser was charged with a mixture of 2-chloro-1-(2,4-disubstituted phenyl) ethanone (**2a**–**e**) (40 mmol) and 1,2,4-triazole (40 mmol, 3.31 g) which refluxed in 40 mL toluene in the presence of sodium bicarbonate (48 mmol, 4.04 g) for 24 h. Then the reaction mixture was poured into crushed ice and extracted with ethyl acetate (2 × 50 mL). The combined organic layers were washed with water and brine, dried over anhydrous sodium sulfate and concentrated in a vacuum. The compounds **3a**–**e** crystallized from diethyl ether.

#### 3.1.3. General Procedure for the Synthesis of 1-((2-(2,4-Disubstituted phenyl)oxiran-2-yl)methyl)-1*H*-1,2,4-triazole (**4a**–**e**)

A mixture of 1-(2,4-disubstituted phenyl)-2-(1*H*-1,2,4-triazol-1-yl) ethanone (**3a**–**e**) in 15 mL dichloromethane, trimethylsulfoxonium iodide, TMSI, (18.03 mmol, 3.97 g) and 30 mL toluene was charged by sodium hydroxide solution (20%, 3.7 mL) and tetraethyl ammonium bromide TEAB (3 mmol, 0.63 g). The reaction mixture was heated for 7 h at 60 °C. Progress of the reaction was monitored by TLC. After completion of the reaction, the product was diluted with water and extracted with ethyl acetate (2 × 40 mL). The organic layer was washed and dried over anhydrous sodium sulfate and concentrated. The crude products were purified by a preparative thin layer chromatography using chloroform and methanol (94:4) to get the brown oily products (**4a**–**e**).

#### 3.1.4. General Procedure for the Synthesis of 2-(2,4-Disubstituted phenyl)-1-(3-nitro-1*H*-1,2,4-triazol -1-yl)-3-(1*H*-1,2,4-triazol-1-yl)propan-2-ol (**5a**–**e**, series A)

Twenty mL of solution of 1-((2-(2,4-disubstituted phenyl)oxiran-2-yl) methyl)-*1H*-1,2,4-triazole (**4a**–**e**) (3 mmol) and triethylamine (1.8–2.9 mL) in absolute ethanol was added to 3-nitro-1,2,4-triazole (4 mmol, 0.456 g) and refluxed for 15–24 h. TLC monitoring was used to control reaction progress. The mixture was filtered, concentrated and diluted with 30 mL water. Then filtrate was extracted with ethyl acetate (3 × 80 mL). The combined organic layers were washed with water and then with brine, dried over anhydrous sodium sulfate and concentrated in a vacuum. The crude products were crystallized from various solvents to gain the products **5a**–**e** (series A).

#### 3.1.5. General Procedure for the Synthesis of 2-(2,4-Disubstituted phenyl)-1-(3-nitro-*1H*-1,2,4-triazol-1-yl)-3-(*1H*-1,2,4-triazol-1-yl)propan-2-ol (**5f**–**j**, series B)

Twenty mL of solution of 1-((2-(2,4-disubstituted phenyl)oxiran-2-yl) methyl)-1*H*-1,2,4-triazole (**4a**–**e**) (3 mmol) and triethylamine (1.8–2.9 mL) in absolute ethanol was added to 4 mmol of 2-(1-pyperazinyl) ethanol and refluxed for 15–24 h. TLC monitoring was used to control reaction progress. The mixture was filtered, concentrated and diluted with 30 mL water. Then filtrate was extracted with ethyl acetate (3 × 80 mL). The combined organic layers were washed with water and then with brine, dried over anhydrous sodium sulfate and concentrated in a vacuum. The crude products were crystallized from various solvents or purified by preparative thin layer chromatography in a solvent system containing chloroform and methanol (88:12) to get the brown oily products **5f**–**j** (series B).

*2-(2,4-Difluorophenyl)-1-(3-nitro-1H-1,2,4-triazol-1-yl)-3-(1H-1,2,4-triazol-1-yl)propan-2-ol* (**5a**). pale yellow solid (34% yield, m.p.: 159–161 °C). ^1^H-NMR (500 MHz, CDCl_3_, ppm): 8.19 (s, 1H, nitrotriazole), 8.02 (s, 1H, triazole), 7.69 (s, 1H, triazole), 7.17 (td, *J* = 8.5 Hz, *J* = 6.45 Hz, 1H, Ar-H), 6.76 (m, 1H, Ar-H), 6.64 (dt, *J* = 8.5 Hz, *J* = 2.3 Hz, 1H, Ar-H), 6.15 (s, 1H, OH), 4.72 (d, 1H, *J* = 14.5 Hz, CH_2_), 4.63 (d, *J* = 12.5 Hz, 2H, CH_2_), 4.54 (d, *J* = 14.5 Hz 1H, CH_2_). ^13^C-NMR (75 MHz, CDCl_3_): δ = 152.42 (C-NO_2_), 145.89 (C_3_ triazole), 144.35 (C-3 triazole, C-5 nitrotriazole), 130.08 (2,4-F2Ph C-1), 112.82 (2,4-F2Ph C-5), 104.94 (2,4-F2Ph C-3), 74.97 (C-OH), 56.87 (CH_2_-nitrotriazole), 54.03 (CH_2_-triazole). MS (*m*/*z*, %): 352 (M^+^ + 1, 16), 269 (51), 224 (100), 132 (11), 155 (29), 141 (87), 127 (100), 113 (19), 101 (11), 83 (100), 55 (58), 41 (11). Anal. Calcd. for C_13_H_11_F_2_N_7_O_3_ (351.3): C, 44.45; H, 3.16; F, 10.82; N, 27.91; O, 13.66. Found: C, 44.25; H, 3.32; N, 27.84.

*2-(2,4-Dichlorophenyl)-1-(3-nitro-1H-1,2,4-triazol-1-yl)-3-(1H-1,2,4-triazol-1-yl)propan-2-ol* (**5b**). White solid (35% yield, m.p.: 168–170 °C). ^1^H-NMR (500 MHz, CDCl_3_, ppm): 8.36 (s, 1H, nitrotriazole), 8.10 (s, 1H, triazole), 7.89 (s, 1H, triazole), 7.58 (d, 1H, *J* = 8.6 Hz, Ar-H), 7.43 (d, 1H, *J* = 1.9 Hz, Ar H), 7.21 (dd, 1H, *J* = 8.6 Hz, *J* = 1.9 Hz, Ar-H), 5.82 (brs, 1H, OH), 5.56 (d, 1H, *J* = 14.4 Hz, CH_2_), 5.10 (d, 1H, *J* = 14.4 Hz, CH_2_), 4.82 (d, 1H, *J* = 14.4 Hz, CH_2_), 4.39 (d, 1H, *J* = 14.4 Hz, CH_2_). ). ^13^C-NMR (75 MHz, CDCl_3_): δ = 152.32 (C-NO_2_), 146.99 (C-3 triazole), 144.18 (C-3 triazole, C-5 nitrotriazole), 136.21 (2,4-Cl_2_ Ph C-1), 133.58 (2,4-Cl_2_ Ph C-4), 131.05 (2,4-Cl_2_ Ph C-2), 130.50 (2,4-Cl_2_ Ph C-3), 130.00 (2,4-Cl_2_ Ph C-6), 128.23 (2,4-Cl_2_ Ph C-5), 76.27 (C-OH), 55.5 (CH_2_-nitrotriazole), 52.57 (CH_2_-triazole). MS (*m*/*z*, %): 384 (M^+^, 10), 348 (8), 301 (10), 256 (33), 214 (100), 187 (11), 173 (25), 158 (43), 145 (11), 123 (13), 109 (16), 82 (85), 55 (45), 42 (8). Anal. Calcd. for C_13_H_11_Cl_2_N_7_O_3_ (384.2): C, 40.64; H, 2.89; Cl, 18.46; N, 25.52; O, 12.49. Found: C, 40.24; H, 2.9; N, 25.32.

*2-(4-Fluorophenyl)-1-(3-nitro-1H-1,2,4-triazol-1-yl)-3-(1H-1,2,4-triazol-1-yl)propan-2-ol* (**5c**). White crystalline solid (35% yield, m.p.: 181–182 °C). ^1^H-NMR (500 MHz, CDCl3, ppm): 8.26 (s, 1H, nitrotriazole), 7.93 (s, 2H, triazole), 7.29–7.7.32 (m, 2H, Ar-H), 7.04–7.08 (m, 2H, Ar-H), 5.52 (s, 1H, OH), 4.71 (d, 1H, *J* = 14.2 Hz, CH_2_), 4.64 (d, 1H, *J* = 14.4 Hz, CH_2_), 4.57 (d, 1H, *J* = 14.4 Hz, CH_2_), 4.40 (d, 1H, *J* = 14.2 Hz, CH_2_). ^13^C-NMR (75 MHz, CDCl_3_): δ = 152.50 (C-NO_2_), 146.78 (C-3 triazole), 144.61 (C-3 triazole, C-5 nitrotriazole), 126.63 (4-FPh C-2,C-6), 116.11 (4-FPh C-3, C-5), 76.57 (C-OH), 58.46 (CH_2_-nitrotriazole), 55.25 (CH_2_-triazole). MS (*m*/*z*, %): 334 (M^+^ + 1, 16), 251 (55), 206 (100), 164 (66), 137 (41), 123 (82), 109 (100), 95 (55), 83 (100), 55 (49), 42 (9). Anal. Calcd. for C_13_H_12_FN_7_O_3_ (333.3): C, 46.85; H, 3.63; F, 5.70; N, 29.42; O, 14.40. Found: C, 46.98; H, 3.91; N, 28.95.

*2-(4-Chlorophenyl)-1-(3-nitro-1H-1,2,4-triazol-1-yl)-3-(1H-1,2,4-triazol-1-yl)propan-2-ol* (**5d**). Pale yellow solid (35% yield, m.p.: 147 °C). ^1^H-NMR (500 MHz, CDCl_3_, ppm): 8.17 (s, 1H, nitrotriazole), 7.92 (s, 1H, triazole), 7.71 (s, 1H, triazole), 7.22–7.29 (m, 4H, Ar-H), 6.08 (brs, 1H, OH), 4.62 (d, 1H, *J* = 14.4 Hz, CH_2_), 4.58 (d, 1H, *J* = 14.3 Hz, CH_2_), 4.49 (d, 1H, *J* = 14.4 Hz, CH_2_), 4.40 (d, 1H, *J* = 14.3 Hz, CH_2 _). ^13^C-NMR (75 MHz, CDCl_3_): δ = 152.51 (C-NO_2_), 147.04 (C-3 triazole), 144.62 (C-3 triazole, C-5 nitrotriazole), 136.96 (4Cl-Ph C-4), 135.20 (4Cl-Ph C-3, C-5), 129.42 (4Cl-Ph C-2, C-6), 126.20 (4Cl-Ph C-1), 75.59 (C-OH), 58.32 (CH_2_-nitrotriazole), 55.17 (CH_2_-triazole). MS (*m*/*z*, %): 350 (M^+^ + 1, 18), 267 (34), 222 (100), 180 (61), 153 (23), 139 (55), 125 (97), 111 (31), 83 (100), 55 (43), 42 (8). Anal. Calcd. for C_13_H_12_ClN_7_O_3_ (349.7): C, 44.65; H, 3.46; Cl, 10.14; N, 28.03; O, 13.72. Found: C, 44.15; H, 3.21; N, 27.91.

*1-(3-Nitro-1H-1,2,4-triazol-1-yl)-2-phenyl-3-(1H-1,2,4-triazol-1-yl)propan-2-ol* (**5e**). White solid (36% yield, m.p.: 160–163 °C). ^1^H-NMR (500 MHz, CDCl_3_, ppm): 8.22 (s, 1H, nitrotriazole), 7.94 (s, 1H, triazole), 7.92 (s, 1H, triazole), 7.31–7.37 (m, 5H, Ar-H), 5.45 (brs, 1H, OH), 4.74 (d, 1H, *J* = 14.2 Hz, CH_2_), 4.68 (d, 1H, *J* = 14.4 Hz, CH_2_), 4.60 (d, 1H, *J* = 14.4 Hz, CH_2_), 4.43 (d, 1H, *J* = 14.4 Hz, CH_2_). ^13^C-NMR (75 MHz, CDCl_3_): δ = 152.10 (C-NO_2_), 145.50 (C-3 triazole), 144.38 (C-3 triazole, C-5 nitrotriazole), 137.92 (Ph C-1), 128.92 (Ph C-3, C-5), 128.70 (Ph C-4), 127.40 (Ph C-2, C-6 ), 75.52 (C-OH), 58.25 (CH_2_-nitrotriazole), 55.18 (CH_2_-triazole). MS (*m*/*z*, %): 316 (M^+^ + 1, 33), 233 (57), 188 (100), 146 (100), 119 (49), 105 (74), 91 (100), 83 (100), 77 (71), 66 (20), 55 (43), 41 (8), 30 (20). Anal. Calcd. for C_13_H_13_N_7_O_3_ (315.3): C, 49.52; H, 4.16; N, 31.10; O, 15.22. Found: C, 49.65; H, 4.32; N, 31.35.

*2-(2,4-Difluorophenyl)-1-(4-(2-hydroxyethyl)piperazin-1-yl)-3-(1H-1,2,4-triazol-1-yl)propan-2-ol* (**5f**). Brown oil (55% yield). ^1^H-NMR (500 MHz, CDCl_3_, ppm): 8.09 (s,1H, triazole), 7.72 (s, 1H, triazole), 7.46–7.49 (m, 1H, Ar-H), 6.73–6.77 (m, 2H, Ar-H), 4.47–4.50 (d, 1H, *J* = 14.3 Hz, CH_2_-triazole), 4.43–4.46 (d, 1H, *J* =14.3 Hz, CH_2_-triazole), 3.50–3.52 (m, 2H, CH_2_-OH), 3.00–3.03 (d, 1H, *J* = 14.1 Hz, CH_2_-piperazine), 2.60–2.63 (d, 1H, *J* = 14.1 Hz, CH_2_-piperazine), 2.41–243 (m, 2H, piperazine-CH_2_), 2.33–2.44 (m, 8H, piperazine), 1.16–1.18 (brs, 1H, OH). ). MS (*m*/*z*, %): 368 (M^+^ + 1, 17.9), 336 (0.7), 299 (1.4), 2.85 (4.3), 143 (100), 127 (5), 113 (3.6), 100 (14.3), 82(3.6), 70 (20.7), 56(5.7). Anal. Calcd. for C_17_H_23_F_2_N_5_O_2_ (367.4): C, 55.58; H, 6.31; F, 10.34; N, 19.06; O, 8.71. Found: C, 55.67; H, 6.25; N, 18.96.

*2-(2,4-Dichlorophenyl)-1-(4-(2-hydroxyethyl)piperazin-1-yl)-3-(1H-1,2,4-triazol-1-yl)propan-2-ol* (**5g**). Brown oil (58% yield). ^1^H-NMR (500 MHz, CDCl_3_, ppm): 8.14 (s, 1H, triazole), 7.78 (s, 1H, triazole), 7.70–7.72 (d, 1H, *J* = 8.5 Hz, Ar-H), 7.36–7.37 (d, 1H, *J* = 2.1 Hz, Ar-H), 7.17–7.19 (dd, 1H, *J* = 2.1 Hz, Ar-H), 4.71–4.74 (d, 1H, *J* = 14.25 Hz, CH_2_-triazole), 4.67–4.70 (d, 1H, *J* = 14.25 Hz, CH_2_-triazole), 3.54 (m, 2H, CH_2_-OH), 3.44–3.47 (d, 1H, *J* = 13.75 Hz, CH_2_-piperazine), 2.67–2.70 (d, 1H, *J* = 13.75 Hz, CH_2_-piperazine), 2.46–2.47 (dd, 2H, *J* = 4.5 Hz, piperazine-CH_2_), 2.33–2.45 (m, 8H, piperazine), 1.20–1.27 (brs,1H,OH). MS (*m*/*z*, %): 400 (M^+^, 8.3), 317 (3.3), 159 (2.5), 143(100), 128 (1.7), 113 (3.3), 100 (15), 82 (3.3), 70 (17.5), 56 (7.5). Anal. Calcd. for C_17_H_23_Cl_2_N_5_O_2_ (400.3): C, 51.01; H, 5.79; Cl, 17.71; N, 17.50; O, 7.99. Found C, 50.97; H, 5.66; N, 17.52.

*2-(4-Fluorophenyl)-1-(4-(2-hydroxyethyl)piperazin-1-yl)-3-(1H-1,2,4-triazol-1-yl)propan-2-ol* (**5h**). Brown oil (42% yield). ^1^H-NMR (500 MHz, CDCl_3_, ppm): 8.01 (s,1H, triazole), 7.73 (s, 1H, triazole), 7.28–7.31 (m, 2H, Ar-H), 6.90–6.93 (t, 2H, *J* = 8.5 Hz, Ar-H), 4.29–4.32 (d, 1H, *J* = 14 Hz, CH_2_-triazole), 4.21–4.24 (d, 1H, *J* = 14 Hz, CH_2_-triazole), 3.47 (t, 2H, *J* = 5 Hz, CH_2_-OH), 2.80–2.83 (d, 1H, *J* = 13.25 Hz, CH_2_-piperazine), 2.55–2.58 (d, 1H, *J* =13.25 Hz, CH_2_-piperazine), 2.36–2.38 (m, 2H, piperazine-CH_2_), 2.21–2.28 (m, 8H, piperazine), 1.10 (brs, 1H, OH). MS (*m*/*z*, %): 350 (M^+^ + 1, 25), 281 (2.14), 267 (3.9), 143 (100), 128 (1.8), 109 (3.9), 100 (12.9), 91(0.7), 82 (2.1), 70 (17.8), 56 (5.7). Anal. Calcd. for C_17_H_24_FN_5_O_2_ (349.4): C, 58.44; H, 6.92; F, 5.44; N, 20.04; O, 9.16. Found C, 58.23; H, 6.74; N, 20.27.

*2-(4-chlorophenyl)-1-(4-(2-hydroxyethyl)piperazin-1-yl)-3-(1H-1,2,4-triazol-1-yl)propan-2-ol* (**5i**). Brown oil (46% yield). ^1^H-NMR (500 MHz, CDCl_3_, ppm): 8.06 (s, 1H, triazole), 7.80 (s, 1H, triazole), 7.26–7.33 (dd, *J* = 9 Hz, 4H, phenyl), 4.32–4.35 (d, 1H, *J* = 14 Hz, -CH_2_-triazole), 4.24–4.27 (d, 1H, *J* = 14 Hz, -CH_2_-triazole), 3.52 (m, 2H, CH_2_-OH), 2.86–2.89 (d, 1H, *J* = 13.5 Hz, -CH_2_-piperazine), 2.62–2.65 (d, 1H, *J* = 13.5 Hz, -CH_2_-piperazine), 2.42–2.44 (dd, 2H, *J* = 3.9 Hz, piperazine-CH_2_-), 2.26–2.34 (m, 8H, piperazine), 1.22–1.30 (brs, 1H, OH). MS (*m*/*z*, %): 366 (M^+^, 18.9), 297 (1.3), 283 (2.5), 143 (100), 125 (3.1), 113 (3.1), 100 (11.9), 91 (0.6), 82(2.5), 70(16.4), 56(6.3). Anal. Calcd. for C_17_H_24_ClN_5_O_2_ (365.8): C, 55.81; H, 6.61; Cl, 9.69; N, 19.14; O, 8.75. Found C, 55.62; H, 6.73; N, 19.03.

*1-(4-(2-Hydroxyethyl)piperazin-1-yl)-2-phenyl-3-(1H-1,2,4-triazol-1-yl)propan-2-ol* (**5j**). Brown oil (38% yield). ^1^H-NMR (500 MHz, CDCl_3_, ppm): 8.02 (s, 1H, triazole), 7.78 (s, 1H, triazole), 7.21–7.38 (m, 5H, phenyl), 4.35–4.38 (d, 1H, *J* = 14.25 Hz, -CH_2_-triazole), 4.25–4.28 (d, 1H *J* = 14.25 Hz, -CH_2_-triazole), 3.50 (m, 2H, -CH_2_-OH), 2.59–2.91 (dd, 2H, *J* = 13 Hz, CH_2_-piperazine), 2.40–2.41 (m, 2H, piperazine-CH_2_-), 2.23–2.31 (m, 8H, piperazine), 1.21–1.24 (brs, 1H, OH). MS (*m*/*z*, %): 332 (M^+^ + 1, 10.7), 249 (6.40), 188 (3.6), 143 (100), 128 (2.9), 113 (3.6), 100 (17.8), 91 (10.7), 82 (5), 70 (26.4), 56 (10). Anal. Calcd. for C_17_H_25_N_5_O_2_ (331.4): C, 61.61; H, 7.60; N, 21.13; O, 9.66. Found C, 61.82; H, 7.52; N, 21.22.

### 3.2. Docking Study

The chemical structures of the compounds were drawn in the Hyperchem 8 and minimized using the AM1 method. Log P for the synthesized compounds and fluconazole were calculated using HyperChem 8. The X-ray crystallographic structure of cytochrome P450 14α-demethylase (PDB deposited code: 1EA1) was retrieved from the protein data bank and was optimized using the Gromacs package [26]. Cognate ligand fluconazole (TPF) was removed from the initial receptor structure and all docking procedures were performed on chain A of 14α-demethylase. All the pre-processing steps for receptor crystallographic structure and partial charge calculations were performed via the AutoDock Tools 1.5.4 program (ADT) (The scripps research institute, La Jolla, California, USA) [27]. To verify the reproducibility of docking calculations, the cognate ligand was redocked, and the final docked conformations resulted in 1–1.5 Å root-mean-square deviation. The Lamarckian genetic algorithm (LGA) was applied to model the interaction between ligands and 14α-demethylase active site. For the Lamarckian genetic algorithm: 27,000 maximum generations; a gene mutation rate of 0.02 and; a crossover rate of 0.8 were used. The grid box size was set to 60 × 60 × 60 points in x, y and z directions, respectively. The box was centered based on the cognate ligand with a spacing of 0.375 Å. Cluster analysis was performed on the docked results using an RMSD (Root Mean Square Deviation) tolerance of 2 Å. All the compounds, as well as fluconazole, were docked according to the aforementioned parameters. Interactions were identified using the LigPlot software and the figures were prepared using VMD 1.9.1.

### 3.3. Biological Activity

Antifungal activities of the synthesized compounds were evaluated against standard and clinical species of the yeasts and filamentous fungi. The compounds were dissolved in DMSO at the concentration of 200 mg/mL. Afterward, compounds were diluted in broth media to obtain final concentrations. Minimal inhibitory concentrations (MIC) of the synthesized compounds were determined by the broth microdilution method as recommended by CLSI (Clinical & Laboratory Standards Institute), with some modifications [28,29]. Briefly, the serial dilutions of compounds **5a**–**j** (625 to 0.5 µg/mL) were prepared in 96-well plates using RPMI 1640 media. Stock inoculums were prepared by suspending three colonies of the tested yeasts in 5 mL of sterile 0.9% NaCl, and adjusting the turbidity of the inoculums to 0.5 at a 530 nm wavelength to yield a stock suspension of 1–5 × 10^6^ cells/mL. For filamentous fungi (*Aspergillus* and dermatophytes), conidia were recovered from 2–7 days old cultures grown on potato dextrose agar by a wetting loop with Tween 20. The collected conidia were transferred in sterile saline and their turbidity was adjusted in OD = 0.09–0.11 (0.4–5 × 10^6^ conidia/mL). Working suspensions were prepared by making a 1/50 and 1/1000 dilution with RPMI of the stock suspension for filamentous fungi and yeasts, respectively. After addition of 0.1 mL of the inoculums to the wells, the trays were incubated at 30 °C for 24–48 h in a humid atmosphere. 200 µL of the uninoculated medium was incubated as a sterility control (blank). In addition, growth controls [medium with inoculums and 5% (*v*/*v*) DMSO (maximum DMSO concentration) without the synthetic compounds] were also incubated. MICs were visually determined and defined as the lowest concentration of the compounds that produced ≥50 growth inhibitions. Each experiment was performed in triplicate and the mean MICs was reported as MIC_50_.

## 4. Conclusions

Here two series of novel fluconazole-derivatives containing nitrotriazole or 2-(piperazin-1-yl) ethanol moieties were synthesized. The antifungal activities of all compounds were evaluated against several standard and clinically isolated yeasts. MICs of the synthesized compounds were obtained and compared with fluconazole. All the synthesized derivatives except **5i**, **5j** and **5h** exhibited moderate to high in vitro antifungal activities. Compound **5b** was the most active antifungal agents against all fungal strains except *Aspergillus* sp; which is comparable to fluconazole. Compounds **5b** and **5g** with two chlorine atoms in their structure showed maximum antifungal activity.

Furthermore, the compounds containing 2-(1-pyperazinyl) ethanol moiety were able to form ester derivatives on the hydroxyl group in ethanol moiety; therefore, these compounds can be considerably prolonged by the preparation of fatty acid esters for a long duration.

## Figures and Tables

**Table 1:** Antifungal activities of **5a**–**5j** against Yeasts (MICs, µg/mL).

Tested Fungi	Compounds										
	5a	5b	5c	5d	5e	5f	5g	5h	5i	5j	Fluconazole
*C. glabrata (CBS 863)*	128	16	G	G	G	256	8	64	G	G	4
*C. dubliniensis (CBS 8501)*	4	0.5	128	16	G	G	32	G	G	G	0.5
*C. dubliniensis (CBS 7987)*	2	0.5	32	4	64	G	1	G	G	G	0.5
*C. dubliniensis (CBS 7988)*	2	0.5	8	2	32	G	32	G	G	G	0.5
*C. albicans (CBS 5982)*	256	128	256	256	G	G	2	G	G	G	256
*C. glabrata (CBS 6144)*	128	32	G	G	G	G	4	G	G	G	4
*C. albicans (CBS 2730)*	16	2	256	32	G	G	64	G	G	G	<0.5
*C. albicans (CBS 562)*	16	1	128	32	G	G	16	G	G	G	<0.5
*C. albicans (CBS 1912)*	8	0.5	128	16	256	16	2	G	G	G	0.25
*C. glabrata (CBS 2192)*	128	32	G	G	G	32	0.5	G	G	G	8
*C. glabrata (CBS 2175)*	128	16	G	G	G	32	1	G	G	G	4
*C. dubliniensis (CBS 8500)*	8	0.5	64	8	256	G	128	G	G	G	0.25
*C. albicans (CBS 1905)*	4	<0.5	64	8	G	G	0.5	G	128	G	0.5
*C. tropicalis (ATCC750)*	16	4	G	64	G	G	4	NT	G	G	2
*C. arapsilosis (ATCC4344)*	16	2	G	128	G	G	8	NT	64	G	0.5
*C. krusei (ATCC6258)*	G	128	G	G	G	64	1	NT	64	G	G
*Cryptococcus neoformance (ATCC9011)*	32	2	256	64	256	G	8	NT	G	G	1
C. albicans *	4	0.5	128	16	256	64	2	256	256	256	1
C. glabrata *	NT	NT	NT	NT	NT	128	8	256	256	256	0.25
C. dublinien *	16	1	256	256	256	16	1	256	128	256	0.5

G: growth, NT: not tested, *: Clinical isolated Yeasts; MICs: Minimal Inhibitory Concentrations.

**Table 2:** Antifungal activities of **5a**–**5j** against filamentous fungi (MIC, µg/mL).

Fungi	Compounds										
	5a	5b	5c	5d	5e	5f	5g	5h	5i	5j	Fluconazole
*Microsporum gypseum*	64	4	G	G	G	256	64	256	G	G	8
*Epidermophyton floccosum*	128	64	G	G	G	64	32	64	G	G	G
*Trichophyton mentagrophytes*	128	128	G	G	G	64	G	G	G	G	G
*Aspergillus fumigatus*	128	G	G	G	G	G	G	G	G	G	G
*Aspergillus fumigatus*	128	G	G	G	G	G	G	G	G	G	G
*Aspergillus fumigatus*	G	G	G	G	G	G	G	G	G	G	G
*Aspergillus clevatus*	G	G	G	G	G	G	G	G	G	G	G
*Aspergillus flavus*	G	G	G	G	G	G	G	G	G	G	G

G: growth.

**Table 3:**
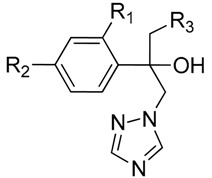
Docking results for compounds **5a**–**j** into the active site of 14α-demethylase enzyme (1EA1).

Compounds	Final Docked Energy (Kcal/mol)	LogP	R1	R2	R3
**5a**	−10.82	0.73	F	F	3-nitro-1,2,4-triazol
**5b**	−11.98	1.48	Cl	Cl	3-nitro-1,2,4-triazol
**5c**	−11.91	0.59	F	H	3-nitro-1,2,4-triazol
**5d**	−11.83	0.97	Cl	H	3-nitro-1,2,4-triazol
**5e**	−11.14	0.45	H	H	3-nitro-1,2,4-triazol
**5f**	−7.17	0.27	F	F	2-(piperazin-1-yl)ethanol
**5g**	−8.26	1.31	Cl	Cl	2-(piperazin-1-yl)ethanol
**5h**	−7.36	0.41	F	H	2-(piperazin-1-yl)ethanol
**5i**	−8.03	0.79	Cl	H	2-(piperazin-1-yl)ethanol
**5j**	−7.25	0.28	H	H	2-(piperazin-1-yl)ethanol
Fluconazole	−6.74	0.99	F	F	1H-1,2,4-triazole
